# Supplementary material for: Perturbations in mitochondrial metabolism associated with defective cardiolipin biosynthesis: An in-organello real-time NMR study
Source: J Biol Chem. 2024 Sep 3;300(10):107746. doi: 10.1016/j.jbc.2024.107746 (PMC11470594; doi:10.1016/j.jbc.2024.107746)
Supplement: Supporting Table and Figures [file mmc1.pdf]

Supporting Material for

Perturbations in mitochondrial metabolism associated with defective cardiolipin biosynthesis: An *in-organello* real-time NMR study

Antonio J. Rua[1], Wayne Mitchell[1#], Steven M. Claypool[2,3,4], Nathan N. Alder[1*], and Andrei T. Alexandrescu[1*]

[1]*Department of Molecular and Cell Biology, University of Connecticut, Storrs, CT 06269, USA*
[2]*Department of Physiology, Johns Hopkins University School of Medicine, Baltimore, MD 21205, USA*
[3]*Mitochondrial Phospholipid Research Center, Johns Hopkins University School of Medicine, Baltimore, MD 21205, USA*
[4]*Department of Genetic Medicine, Johns Hopkins University School of Medicine, Baltimore, MD 21205, USA*
[#]*Current address: Division of Genetics, Department of Medicine, Harvard Medical School, Brigham and Women's Hospital, Boston, MA 02115, USA*

Table of contents:

Table S1 – Metabolite YMDB accession codes, chemical shifts, and structures
Supplementary Figure S1 – Uncropped Tom70 western blots
Supplementary Figure S2 – Mitochondrial respirometry
Supplementary Figure S3 – NMR correlations used to verify metabolite assignments

Table S1: Metabolite YMDB accession codes, chemical shifts, and structures

Metabolite	YMDB ID	Peak Number	^{1}H Shift (ppm)	^{13}C Shift (ppm)	C Atom	Structure
Pyruvate	175	1	2.37	29.37	1	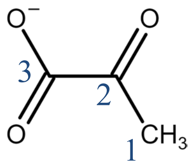
Pyruvate Tautomer		1t	1.49	28.08	1	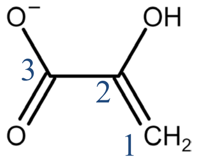
Mevalonate	707	2	1.34	28.05	6	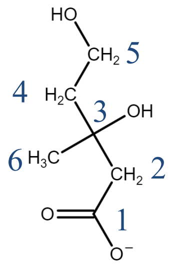
Citramalate	1584	3	1.23	27.23	5	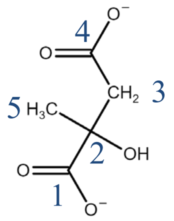
2-isopropylmalate	1487	4<br>7<br>11	0.91<br>0.86<br>1.87	18.73<br>20.13<br>37.70	6<br>6’<br>5	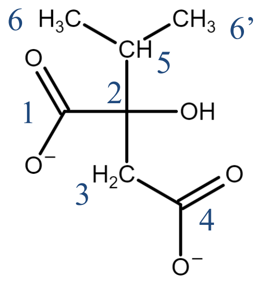
Acetoin	410	5	1.36	19.32	3	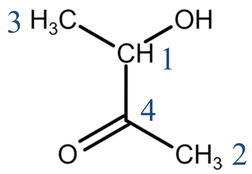

Valine	152	6<br>8	0.95<br>1.00	19.19<br>19.52	4<br>4’	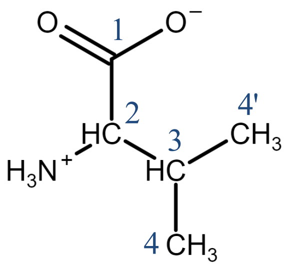
Acetate	59	9	1.93	26.13	2	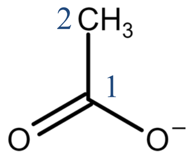
Glutamate	271	10	2.34	35.41	4	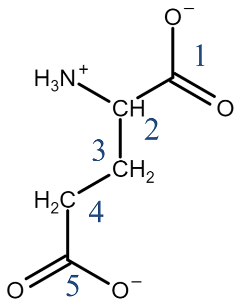
Citrate	86	12	2.68	47.89	1,3	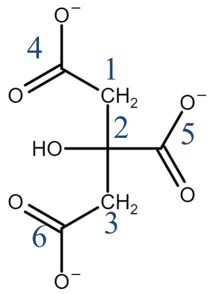
α-ketoisovalerate	365	13<br>15	2.94<br>1.13	39.85<br>19.23	1<br>2,2’	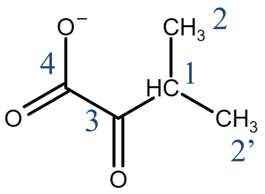

α-ketoglutarate	2	14	2.44	33.51	4	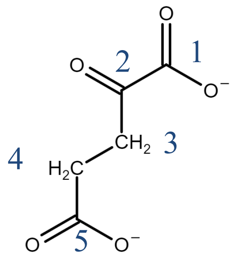
Acetolactate	394	16<br>17	1.47<br>2.26	24.20<br>27.27	3<br>1	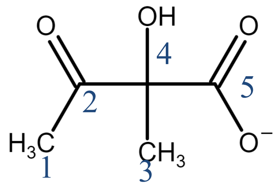
Succinate	338	18	2.41	37.36	2, 3	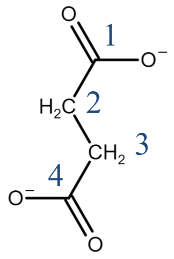

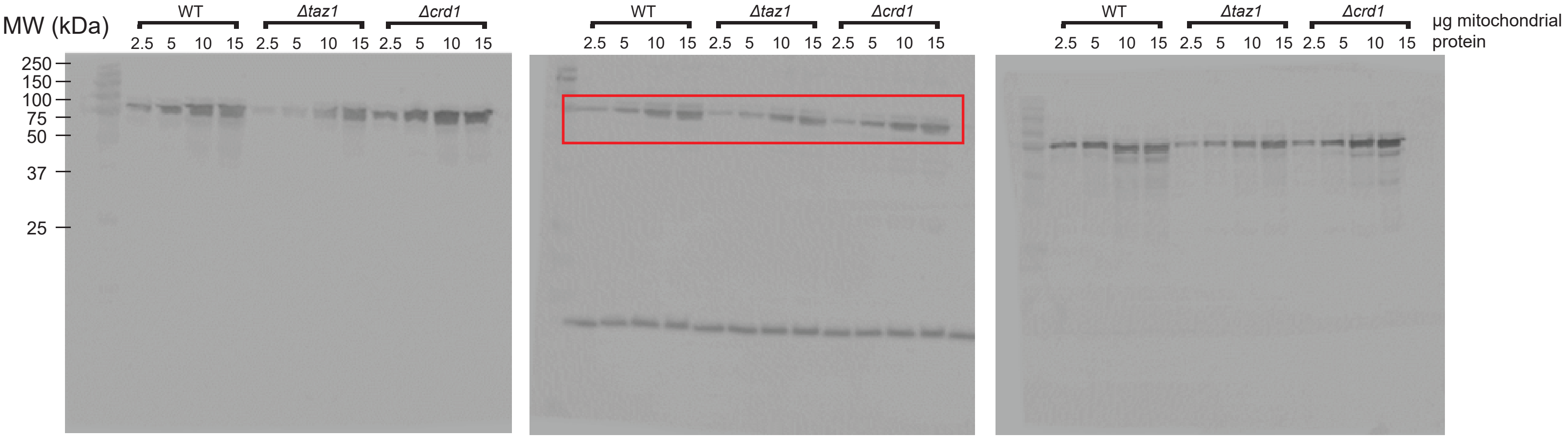

Supplementary Figure S1: Uncropped Tom70 western blots

Uncropped Western blots of Tom70, used for quantification in Figure 2B. Red box indicates cropped image used as representative blot in Figure 2A.

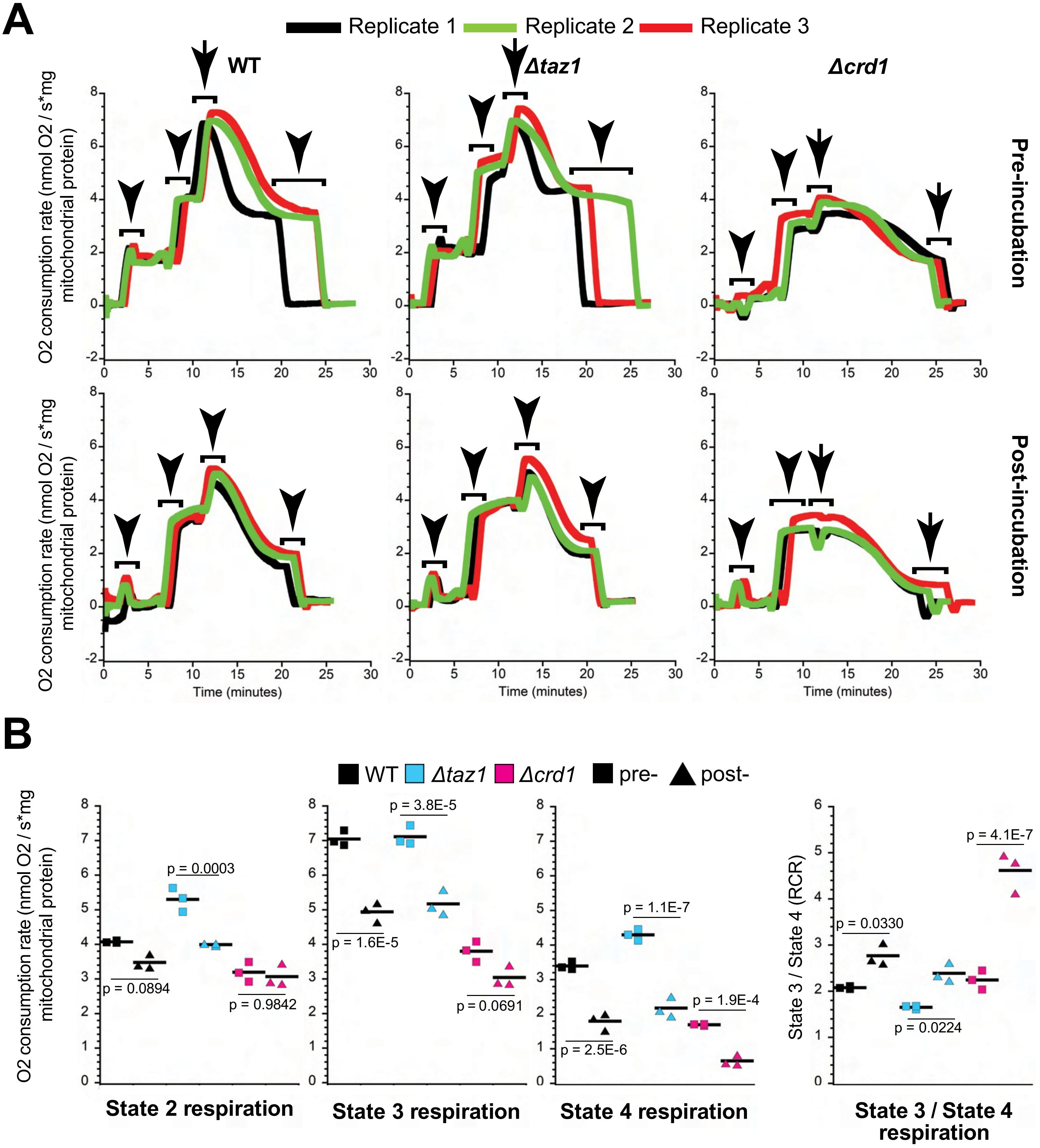

Supplementary Figure S2: Mitochondrial respirometry

(A) Traces from 3 independent biological replicates pre- (top) and post- (bottom) 65 minutes of incubation at 30°C. Arrows represent sequential additions of mitochondria (first arrow), respiratory substrates (malate, pyruvate, and NADH; second arrow), ADP (third arrow), and antimycin A (fourth arrow). *(B) Quantification of respiration rates pre- and post-65 minutes of incubation at 30°C.* State 2 respiration rates were obtained following addition of respiratory substrate (malate, pyruvate, and NADH), State 3 respiration rates were obtained following addition of ADP, State 4 respiration rates were obtained as respiration rates stabilized to a new steady-state after ADP phosphorylation, and respiration was stopped by the administration of antimycin A. p-values were determined by one-way ANOVA and Tukey's post-hoc test.

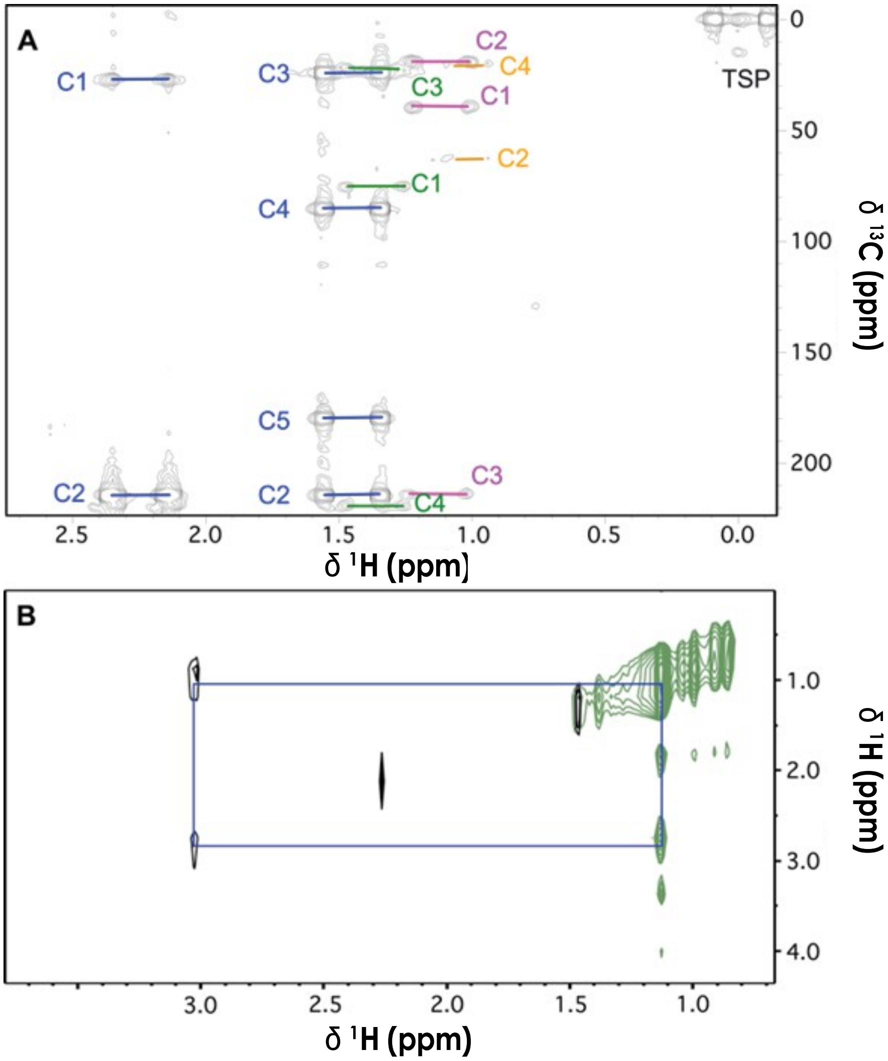

Supplementary Figure S3: NMR correlations used to verify metabolite assignments

(A) HMBC spectrum showing long-range couplings that establish carbon frameworks for acetolactate (blue), acetoin (green), αKI (pink), and valine (orange). Please refer to Table S1 for the carbon atom numbering. *(B) Superposition of ^{13}C planes from a 3D ^{13}C HSQC-TOCSY experiment: 19.1 ppm, green; 41 ppm, black.* The symmetric TOCSY cross peaks in both of the superposed planes connect the αKI C1 and C2 carbons (Table S1) and indicate both sites have ^{13}C-label incorporated.
